# A proposal for teaching bioethics in high schools using appropriate visual education tools

**DOI:** 10.1186/s13010-018-0064-1

**Published:** 2018-07-20

**Authors:** Chiedozie G. Ike, Nancy Anderson

**Affiliations:** 1 Department of Public Health, Irrua Specialist Teaching Hospital, Irrua, Edo State Nigeria; 2 Department of Midwifery, Bastyr University, Kenmore, Washington USA

**Keywords:** Stereotypes, Bioethics, High school, Pedagogy, Media, Visual tools, Teens

## Abstract

Teaching bioethics with visual education tools, such as movies and comics, is a unique way of explaining the history and progress of human research and the art and science of medicine to high school students. For more than a decade, bioethical concepts have appeared in movies, and these films are useful for teaching medical and research ethics in high schools. Using visual tools to teach bioethics can have both interpretational and transformational effects on learners that will enhance their overall understanding of complex moral and legal issues in medicine and research.

High school students are uniquely suited to learn bioethics because they will soon become legal adults. As adults, they will make moral decisions that may affect their health and wellbeing as well as that of their communities and societies.

However, not all visual education tools are appropriate for bioethics pedagogy in high school. Bioethics film and comic producers must consider the specifics of student age, race, gender, belief, level of education, and sexual orientation. Such tools must not be dominated by either dystopic or utopic genres, must aim for objectivity, and must consider the complexity of ethical decision making. It is critical that the teacher, who is the final arbiter regarding the use of visual tools in the classroom, determines that the visual learning tool is acceptable for students in any particular education context. In addition, during the conceptualization and creation of these tools, bioethics film and comic producers must work harder to ensure that these visual tools are devoid of any form of stereotyping.

## Background

The use of visual education tools, such as films and comic strips, in teaching bioethics is an excellent method of presenting new or complicated moral and philosophical ideas to high school students who, for this paper, include persons between 14 and 18 years of age who are expected to be in high school. Bioethics movies and comics are unique ways of explaining the history and progress of the art and the science of curative treatments, the physician-patient relationship, and human and other forms of biological experiments. The transformation of text into visual education tools can help teenagers understand and remember arguments and viewpoints about complex ethical issues such as animal experiments, biological enhancements, organ donation, surrogacy, gene technologies, reproductive technologies, and the end of life. In the context of this article, these tools can include interactive pictorials, motion pictures, cartoons and illustrated story books. The immediate engagement associated with visual tools is also a powerful method of persuasion. Compared with the dystopic framework and abundant negative stereotyping in mainstream media, a visual learning tool for classroom bioethics must be able to strike a balance between the real world and theatrical performance while preserving the content of these complex ethical issues. Acceptable visual bioethics education tools must avoid any form of negative labelling of any group of people, culture, tribe, gender, or class. This paper, therefore, examines and supports the relevance of appropriate films, comics and illustrated story books for teaching bioethics to high school students.

As a general introduction, the following quote from Solbakk [1] elucidates the importance of illustrations in Bioethics as a whole:

“*Since its very origins, cinema has promoted awareness of ethical problems as problems that not only affect the minds of people, but they engage their appetites, beliefs, emotions, and desires as well. Thus, through the cinematic medium ethics emerges as a discipline genuinely and profoundly entangled in the human affairs of this world. With the expansion of the film industry, this way of representing moral conflicts has reached wider audiences and promoted interesting discussions inside and outside the academic world. With the creation of digital technology, many people are participating in a renewed wave of cinematographic passion.”*

## Why teach bioethics to high school students?

What is high school and why should bioethics be introduced into the school curriculum? In this article, high school is defined as school grades 9–12 (ages 14–18) [2]; it is the time of transition between childhood and adulthood, the age of adolescence [3]. It is a period when an individual may accept or reject any sociocultural identity or expression. Teens are exposed to media stereotypes about human beings and about a world context that may veer between dystopia and utopia. On average, they are exposed to media stereotypes by the time they graduate from middle school [4]. During this period, teenagers form personal opinions about themselves and their societies. For most teenagers, this stage is their first time to make a moral decision without the involvement of their parents, caregivers or teachers in the decision-making process. It is a very crucial age in their lives; if not properly handled, when they become full citizens or legal adults, they may not be able to make rational decisions related to their own lives, their communities, or the political system in their overall society. For high school students to become ideal decision makers in politics, education, health care, research, and social welfare, it is crucial that they be taught problem-solving or life-coping skills that will enable them to make ethical decisions impacting not only their lives but also the lives of others.

Arguably, high school students are best suited to learn bioethics because they are at a developmental stage when their cognitive, emotional and physical development are all coming together [5]. In a matter of less than six years following middle school, and in most nations of the world, they will be regarded as legal adults that have the right to give consent to participate in any event [6]. They will also be permitted by law to express their opinion about health care, political, religious, educational and sociocultural systems without the permission of their parents or caregivers. In high school, students may come face to face, for the first time, with bioethical topics such as organ donation, eugenics, animal experimentation, surrogacy, assisted reproduction, physical enhancement, and the philosophies of science, religion, and life. These grades, therefore, constitute a very critical period. Thus, the high school period should, conceivably, be the best time to formally introduce lessons in ethics to the school curriculum.

## Visual learning tools in classroom learning

Visual learning tools such as motion pictures and comics have been in the classroom for decades [7]. Some studies have associated the use of visual learning tools with increased reading literacy and the ability to teach diverse science topics [7–12].

Outside of the classroom, visual tools also educate. They may help patients understand and cope with the complicated symptoms of their health conditions [13]. Visual tools as narratives to accompany health communication have the potential to facilitate attention, comprehension, and recall of embedded messages [14]. For example, the use of comics in a classroom may help to open up discussions about very sensitive and challenging topics such as life following a donor conception, what it means to be a child from surrogate pregnancy, the making of ‘designer babies,’ euthanasia, deception in research and abortion.

In theory, visual illustration, when used to accompany a text, can have interpretational and transformational effects [15]. These effects help to clarify difficult texts and assist in enhancing memory. Thus, texts accompanied by illustration can attract attention, aid in retention, improve understanding or create context [16].

Comics and movies in pedagogy can also enhance the cognitive skills necessary to develop and organize ideas and relationships and to categorize concepts. The tools aid students in the retention and recall of information. Karla, considering the advantage of illustrations over texts [17], asserts that courses that use graphic interfaces consisting of photos, illustrations, charts, maps, diagrams, and videos are gradually replacing text-based courses. In their article “Are Comic Books an effective way to engage non-majors in learning and appreciating science?” Hosler and Boomer [18] suggest that comic book stories can play a significant role in coherently conveying content, while also improving the attitudes of non-science majors towards biology. These authors wanted to develop a strategy to address the low level of science literacy in America by communicating the fascination, joy, and utility of science [18]. They used pre- and post-teaching assessment to measure students’ attitudes to biology and comics. They also assessed students’ content knowledge about evolution before and after using the science comic book *Optical Allusions* in their classes. Hosler and Boomer observed that when they employed texts, non-majors had the lowest scores on the content test and attitude survey compared to other groups. However, there was a statistically significant improvement in non-major content scores when the illustrated text was used, including scores focused on Sensory Biology (*p < 0.*0001), Organic Evolution (*p < 0.*0313), Neurobiology (*p* = 0.0001), and Biology II (*p < 0.*0001). Attitude scores also improved in Sensory Biology (*p =* 0. 0128 and 0.0104) and Organic Evolution (*p* = 0.0039 and 0.0313) after using the post-instruction active intervention instrument, a biology comic book. Investigators also observed that individual student attitudes about biology were positively correlated with their attitudes about comics. In conclusion, they stated that comic book stories are not inferior to traditional textbooks while having the additional potential benefit of improving attitudes about biology.

## Visual narratives are not new in bioethics pedagogy

Using the media to communicate health issues either in the class room or inside hospital wards is no longer news. Generally, the media provide enormous amounts of information about health through story lines in entertainment programming that are accessible to adolescents [19]. There is potential for these pedagogical tools to facilitate attention to and comprehension and recall of embedded messages when intended learners have difficulty processing a more didactic message. This potential may have already inspired their application in the teaching of bioethics and other complex health topics. The importance of teaching bioethics to high school students has already prompted several institutions, such as the National Institutes of Health [20], the North West Association of Biomedical Research [21], Georgetown University [22], and Saint John Paul the Great Catholic High School [23] to teach bioethics to high school students and to also employ communication entertainment education as part of schools’ Bioethics Projects. Some academic institutions have excellent visual resources available for learning bioethics. There are more than 700 films, including rare, hard-to-find movies, at the Georgetown University Bioethics Research Library, which may be the best source of films with a bioethics focus or interpretation [24]. In the last two decades, some movies have been specifically chosen to open up conversations in bioethics classes. A few of the movies and what they illustrate are highlighted below:

Over a period of three academic years (2007–2010), Magi and Jorge [25] used three films to promote the learning of the principles of bioethics. Their other purpose was to stimulate discussion about the issues in their bioethics syllabus. The three movies were selected based on their commercial success and their ability to stimulate discussion on a particular topic. They picked *The Doctor (1991),* directed by Randa Haines, to illustrate the doctor-patient relationship. *Miss Evers’ Boys* (1997), directed by Joseph Sargent, explained the history behind the formulation of the basic principles of bioethics and presented the ethical issues related to human experiments. *Extreme Measures* (1996), directed by Michael Apted, illustrated proper scientific practice and the limits of research. In their study, Mega and Jorge observed a positive response to the use of films from the learners, as measured by their level of classroom participation.

In their study, Mega and Jorge showed that it is possible to teach bioethics using visual tools such as movies. However, the three films employed by Mega and Jorge might be considered inappropriate for some high school audiences due to the negative stereotypes and their dystopic content, as discussed in detail in the subsequent sections.

## Some films and comic strips may not be appropriate

In this proposal, the potential for visual tools to facilitate attention and comprehension and to open up a discussion in bioethics has been stated clearly in the preceding section. However, for visual tools, such as comics and movies, to function as appropriate learning tools in bioethics, both the creators of those visuals and the teachers that plan to use them in the classroom should consider the degree of dystopian or utopian conceptualization and the stereotypes seen in movies and comics. It is also critical that schools evaluate the level of media literacy of each learner before adding the tools to school pedagogy.

## Movies and comics from utopic or dystopic genres: How do they relate to bioethics education?

The selection criteria for classroom media tools must include an analysis of the utopic or dystopic bias portrayed or described. In a movie or comic characterized by utopia, the context is idealized to include peaceful government, equality of citizens, access to education, healthcare, employment, and a safe environment [26]. The entire storyline shows a world where there is no trouble, where there is universal happiness and where everything works, an unrealistic wonderland. On the other hand, abundant imperfections and problems characterize dystopic genres in films and literature. While utopic fiction casts a false picture of a present or near-future paradise on earth, dystopic genres warn the reader or viewer of an impending catastrophic judgement or apocalypse. Dystopic fiction paints a bleak future where timely intervention from “a hero” or “a chosen one” must occur to prevent utter destruction. The disparate interests of young people in the dystopic genre can be a source of concern because of the violent content and pessimistic outlook of the genre, though proponents of dystopic movies have defended the style as realistic and accurate to the real world [27]. Those who support dystopic media believe that it can help the viewer prepare for present or future harsh realities [28].

It is not immediately apparent why the majority of bioethics movies and comic books are dystopic in style. The dystopic genre may be prevalent in bioethics films because dystopia is more reflective of the real world. The most common context for bioethical dilemmas is dystopian, in the sense that deliberate wrongdoing, ignorance, and injustice often lead to bioethical shortcomings. Additionally, current and well-communicated world crises may be connected to the commercial success of the dystopian genre. Dystopian media are frequently more commercially successful, attract more users, may be more immediately engaging, and more able to catalyse class discussion than utopic narratives. Given that the dystopic genre often inflates the dystopia in the real world, creators or makers of bioethics media for pedagogical purposes need to be mindful of the exaggeration that often obscures the truth in commercial media. A bioethics movie that is appropriate for classroom use should therefore narrate events accurately. Appropriate films require more than the masterly performances or viewer engagement that lead to commercial success.

In sum, an appropriate visual tool should be realistic without exaggerating reality as popular genres do. The visual tool should have a balance between today’s harsh realities and a hopeful tomorrow. In addition, entertaining films or comics to be used as education tools should be created and chosen based on the recognition that what may be dystopic to a particular student or user may be utopic to another. Media choices that reflect the flaws in historic and current events as well as the potential for justice and restitution will be the most appropriate visual tools to illustrate the complexities of a moral subject.

## Films and comics containing negative stereotypes

The literature provides many examples of stereotyping in todays’ mainstream media as well as potential effects on adolescents [29–34]. Negative labelling has a lasting detrimental impact on those who experience it [35]. Common stereotypes with respect to race, culture, religion, ethnicity, socioeconomic status, gender, sexual orientation, are embedded, both consciously and unconsciously, in mainstream media today. At least one study has demonstrated that the lingering effects of negative stereotypes may include aggressive behaviour, lack of self-control, difficulty with rational decision making and overindulgence in unhealthy foods.^36^ The findings suggest that there are long-term consequences for individuals who must cope with negative stereotypes. For high school students, this means that they may become adults whose attitudes, values and self-esteem are shaped by these effects. It is therefore vital that any visual tool that accompanies high school bioethics education be devoid of negative stereotypes. Where it is feasible, bioethicists, educators, and other stakeholders in high school pedagogy should engage with the creators of mainstream narratives, such as those who develop Hollywood movies, Disney cartoons, Marvel and DC comics. This interdisciplinary collaboration will be able to produce stories that illustrate a topic in bioethics and produce a desired effect on classroom learning without overtly or covertly profiling any group, gender, tribe or culture. A successful effort at developing accurate and fully dimensioned visual narratives will enforce the over-arching purpose of bioethics in high school: to impart knowledge and skill that will help adolescents make rational decisions in adulthood.

## Conclusion

High school students are uniquely suited to learn bioethics because they are preparing for independent lives. As adults they will make decisions that will affect not only them but also their community and society as a whole.

To enhance the understanding of sensitive and challenging topics in bioethics in a high school class, visual tools such as comics and films should be employed for their potential to facilitate attention to and comprehension and recall of embedded messages and for their ability to engage young minds.

However, for a visual tool to be appropriate for bioethics pedagogy, its content and style should be designed to not distort or exaggerate facts, and it should not negatively profile any people, culture, gender, or group.

High school educators should consider analysing their current collections of bioethics films and comics for possible exaggeration and profiling with the aim of subsequently discarding inappropriate content. Institutions should explore the possibility of collaborating with film makers and comic writers/illustrators to broaden and scale up production of appropriate visual tools for the teaching of bioethics in high schools.
